# Supercritical Fluid CO_2_ Extraction and Microcapsule Preparation of *Lycium barbarum* Residue Oil Rich in Zeaxanthin Dipalmitate

**DOI:** 10.3390/foods10071468

**Published:** 2021-06-24

**Authors:** Yan Men, Shaoping Fu, Chao Xu, Yueming Zhu, Yuanxia Sun

**Affiliations:** 1 National Engineering Laboratory for Industrial Enzymes, Tianjin Institute of Industrial Biotechnology, Chinese Academy of Sciences, Tianjin 300308, China; men_y@tib.cas.cn (Y.M.); fu_sp@tib.cas.cn (S.F.); xuchao@tib.cas.cn (C.X.); zhu_ym@tib.cas.cn (Y.Z.); 2 National Technology Innovation Center of Synthetic Biology, Tianjin 300308, China

**Keywords:** *Lycium barbarum*, supercritical fluid CO_2_ extraction technology, carotenoid, microencapsulation

## Abstract

The scope of this investigation aimed at obtaining and stabilizing bioactive products derived from *Lycium barbarum* seeds and peels, which were the byproducts in the processing of fruit juice. Zeaxanthin dipalmitate is a major carotenoid, comprising approximately 80% of the total carotenoid content in the seeds and peels. The method of obtainment was supercritical fluid CO_2_ extraction, studying different parameters that affect the oil yield and content of zeaxanthin dipalmitate. The optimized protocol to enact successful supercritical fluid CO_2_ extraction included optimum extraction pressure of 250 bar, temperature at 60 °C over a time span of 2.0 h, and a CO_2_ flow of 30 g/min, together with the use of a cosolvent (2% ethanol). The yields of oil and zeaxanthin dipalmitate under these optimal conditions were 17 g/100 g and 0.08 g/100 g, respectively. The unsaturated fatty acids were primarily linoleic acid (C18:2), oleic acid (C18:1), and γ-linolenic acid (C18:3), with their contents being as high as 91.85 ± 0.27% of the total fatty acids. The extract was a red-colored oil that was consequently microencapsulated through spray-drying with octenylsuccinate starch, gum arabic, and maltodextrin (13.5:7.5:3, *w*/*w*) as wall materials to circumvent lipid disintegration during storage and add to fruit juice in a dissolved form. The mass ratio of core material and wall material was 4:1. These materials exhibited the highest microencapsulation efficiency (92.83 ± 0.13%), with a moisture content of 1.98 ± 0.05% and solubility of 66.22 ± 0.24%. The peroxide content level within the microencapsulated zeaxanthin dipalmitate-rich oil remained at one part per eight in comparison to the unencapsulated oil, following fast-tracked oxidation at 60 °C for 6 weeks. This indicated the potential oxidation stability properties of microcapsule powders. Consequently, this microencapsulated powder has good prospects for development, and can be utilized for a vast spectrum of consumer health and beauty products.

## 1. Introduction

*Lycium barbarum* (*L. barbarum*), which is known as Gouqizi or Chinese wolfberry in China, is a traditional Chinese medicine widely utilized for liver and eyesight protection and as an antioxidant. It is also a common ingredient in tonic food due to its promising antiaging and anticancer preventive roles, cardiovascular protection, and capacity to restore immune-system functionality. The increasing production of *L. barbarum* juice and other processed products has significantly elevated the issues of environmental pollution and resource waste due to the accumulation of a large amount of *L. barbarum* waste. The content of waste residues, such as peels and seeds, accounts for 20–25% of the total fresh fruit of *L. barbarum* [1].

Previous studies demonstrated that a large number of carotenoids, especially zeaxanthin dipalmitate, which accounts for >56–75% of the total pigment content, accumulate in the ripe fruits of *L. barbarum*, which also contain small amounts of zeaxanthin, zeaxanthin monopalmitate, lutein, carotene, and β-cryptoxanthin [1,2]. Zeaxanthin dipalmitate, which has many conjugated double bonds, consists of a yellow-coloured pigment and is a lutein isomer, together with being a β-carotene constituent. Upon ingestion, zeaxanthin dipalmitate is accumulated within adipose tissues, particularly in the retinal macula. Studies revealed that zeaxanthin aids in UV-induced macular degeneration prophylaxis [3,4,5]. Yang et al. [6] quantified zeaxanthin dipalmitate and overall carotenoid content within *Lycium* fruits (*Fructus lycii*). The study reported that there existed an overall range of 0.03–0.5% total carotenoid content in various *Fructus lycii*, with zeaxanthin dipalmitate being the predominant carotenoid, consisting of 31–56% of all carotenoid content in *Fructus lycii*. *L. barbarum* seeds also contain abundant oils, with over 80% consisting of unsaturated fatty acids such as linolenic, oleic, and linoleic acids. *L. barbarum* seed oil was found to exhibit highly effective antioxidant roles [7,8]. Therefore, such an oil has the potential for widespread application within the health and beauty consumer product market, while its successful microencapsulation-based stabilization also could make this oil useful within the food industry [9].

*L. barbarum* seed oil has been traditionally extracted through mechanical and/or chemical segregation processes. However, mechanical processes typically have low content yields, while chemical processes that use solvents such as chloroform, hexane, and petroleum ether are all biohazardous, and additionally can pollute the environment. Consequently, supercritical fluid CO_2_ extraction (SFE-CO_2_) is an emerging food-processing technology since it is nonexplosive, nontoxic, environmentally friendly, cost-effective, and time-saving. When polar compounds are targeted, low volumes of organic solvents are required [10,11].

Conversely, increased susceptibility to oxidative damage and eventual loss of desirable flavors is a predominant issue with oils rich in polyunsaturated fatty acids. Therefore, it is necessary to protect these oils to make them more stable during handling, processing, and storage [12]. Microencapsulation technology endows prophylaxis for environmentally induced oil degradation through the shifting of the nature/performance of the actual material, allowing the protection of unique odors, enhancing the stability of such oils, and ultimately extending product shelf-life. In order to enhance oil stabilization and consequently extend potential application ranges, a specific formula can be developed for performing oil microencapsulation through spray-drying [13].

In the present study, we used the residues (primarily seeds and peels) of *L. barbarum* as raw materials and applied SFE-CO_2_ technology to extract the seed oil of *L. barbarum*. The optimum processing conditions were obtained using the single-factor method. Overall, seed oil carotenoid presence and fatty acid integrity were analyzed. In order to enhance *L. barbarum* seed oil shelf life and to expand the scope of its processing and application, the spray-drying method was adopted to investigate microcapsules and analyze the oxidation resistance of microcapsule products.

## 2. Materials and Methods

### 2.1. Materials and Chemicals

*L. barbarum* dry residues (seeds and peels) were collected from Ningxia Hui Autonomous Region, China. The reference compound zeaxanthin dipalmitate (alternate name: physalien) was purchased from Shanghai Yuanye Biotechnology Co., Ltd. (Shanghai, China), while its purity (≥95%) was determined with high-performance liquid chromatography-diode array detection (HPLC-DAD). The lutein standard was purchased from Sigma Chemical Company (St. Louis, MO, USA). The wall materials for microencapsulation, including gum arabic, maltodextrin, and soy protein isolate, were purchased from Shanxi Sciphar Natural Products Co. Ltd. (Xi’an, Shanxi, China). Octenylsuccinate starch (OSA-starch) was purchased from Wanbo Chemical Products Co. Ltd. (Zhengzhou, Henan, China). Polysaccharides were extracted from *L. barbarum*. The methyl-tert-butyl ether (MTBE) and methanol (MeOH) were purchased from Merck (Darmstadt, Germany). Analytical grade reagents were also utilized in this study.

### 2.2. Preparation of Zeaxanthin Dipalmitate-Rich Oil

#### 2.2.1. Preparation of Extraction Process

The particle size of a *L. barbarum* seeds and peels mixture can influence the extraction efficiency of SFE-CO_2_ and reduce the particle size of the sample, which can increase the contact surface area of the mixture, thereby improving the extraction efficiency. However, when the particle size is too small or fine, they may block the filter at the outlet of the equipment extractor. In this experiment, it was more appropriate to crush *L. barbarum* seeds and peels to an 80–100 mesh size. The presence of water in *L. barbarum* seeds and peels severely obstructed the contact between water-insoluble SFE-CO_2_ and hydrophobic carotenoids, which significantly affected the extraction efficiency of carotenoids. In the experiment, it was advisable to bake the *L. barbarum* seeds and peels mixture powder in a 55 °C oven to achieve a fixed weight and then calculate the moisture content. [14].

#### 2.2.2. SFE-CO_2_ Extraction and Protocol Optimization

Optimizing the SFE-CO_2_ extraction protocol variables for *L. barbarum* residues was at the core of the entire process, and was also the most important factor in determining the extraction rate of zeaxanthin dipalmitate. During the study, the extraction pressure, CO_2_ flow rate, temperature, and timing were varied to establish an efficient extraction process for the *L. barbarum* pigment residues.

SFE-CO_2_ extraction treatment: in all experiments, 50 g samples (mixture of *L. barbarum* seeds and peels) prepared as described above were placed in the extractor autoclave, and after an initial air purge, liquefied CO_2_ was pumped into the vessel, and the pressure consequently was raised. At the end of each treatment, the pressure was quickly released within 1 min. The extraction temperatures were adjusted to 40 °C, 50 °C, 60 °C, and 70 °C. Pressure levels were adjusted to 150, 200, 250, 300, and 350 bars, while the cosolvent consisted of 1.0%, 2.0%, and 5.0% ethanol (*w*/*w*, g/100 g biomass introduced in the extraction autoclave), which was pumped into the extraction autoclave with extraction time. The CO_2_ flow rate was set to 15, 20, 25, 30, and 35 g/min. The maximum time of extraction was 2.5 h, when saturation in the extraction curve was observed. The optimum conditions for SFE-CO_2_ extraction were established according to the extract yield and total zeaxanthin dipalmitate content collected from *L. barbarum* residues.

The extract was collected in a flask connected to the back-pressure regulator and consequently stored at −20 °C prior to further analysis to determine the extract yield and bioactive components. The following equations were used in the analysis:

Oil yield (%) = mass of extracted oils/mass of the biomass introduced in the extraction autoclave × 100%(1)

Zeaxanthin dipalmitate yield (%) = mass of extracted zeaxanthin dipalmitate/mass of the biomass introduced in the extraction autoclave × 100%(2)

#### 2.2.3. Quantification of Zeaxanthin Dipalmitate and Total Carotenoids

The concentration of zeaxanthin dipalmitate and total carotenoids was quantified with HPLC using a YMC^TM^ C30 carotenoid column (250 mm × 4.6 mm D.S-5 μm) (Shimogyo-ku, Kyoto, Japan) equipped with a UV detector operating at 450 nm [15]. The most appropriate solvent system was found to be composed of methanol (MeOH) 100% (A, equilibrium phase) and methyl tertbutyl ether (MTBE) 100% (B, elution phase) with the following gradient elution: 86% A and 14% B in 10 min initially, increased to 30% B from 10–20 min, then 50% B from 20–40 min, then kept at 50% B from 40–60 min, and returned to 14% B at 65 min. The flow rate was 1 mL/min at 30 °C. The samples injected measured 10 μL. External calibration was constructed by using the carotenoid standards. Five amounts (range of 10–100 μg) of lutein, zeaxanthin, and zeaxanthin dipalmitate were injected into HPLC (each standard was dissolved in 1 mL of MTBE, and the injection volume was 10 μL); the linear regression equation for each standard curve was obtained by plotting the amount of standard compound injected against the peak area. The regression equation and correlation coefficient (R^2^) were calculated using Microsoft Excel software 2019.

#### 2.2.4. Analysis of Fatty Acids

The fatty acid methyl esters (FAMEs) of the total lipid fraction were obtained by transesterification at room temperature in capped screw-top tubes, as reported in Blasi et al. [16,17]. Hexane (1 mL) and 2 N KOH in MeOH (0.3 mL) were added to the collected oil of *L. barbarum*; after 3 min, deionized water was added, while the upper organic phase was dried over anhydrous Na_2_SO_4_. Consequently, it was injected into a high-resolution gas chromatography column. Analysis of the FAMEs was performed in an Agilent 7890A GC/7200 Q-TOF MS (Agilent Technologies Inc, Santa Clara, CA, USA) equipped with a split–splitless injector and with an FID (Agilent Technologies Inc, Santa Clara, CA, USA). The separation was obtained using the Agilent J&W DB-5ms column (30 m × 0.250 mm × 0.25 μm). The injector and detector temperature were 280 °C and 325 °C, respectively. The oven temperature was 120 °C held for 1 min, then increased to 325 °C at 20 °C/min; the final temperature was held for 20 min. For the split–splitless injection, the split speed was 100 mL/min, the carrier economizing mode was opened at 5 min, and the purging flow rate was 3 mL/min. The carrier gas (He) flow rate was 1.0 mL/min. The following MS parameters were used: ion source temperature, 300 °C, MS quard 150 °C; detector voltage, 0.9 kV; acquisition mass range, 30–550 u; scan speed, 1000 u/s; solvent delay, 1 min. According to the MS information obtained by GC-MS detection, the database NIST2020 was used for retrieval, and analysis software Masshunter 10.0 was applied. The internal standard was glyceryl triheptadecanoate (C17:0). The mass correction factors of methyl palmitate, methyl stearate, methyl oleate, methyl linoleate, and methyl linolenic acid with respect to methyl glyceryl triheptadecanoate were calculated. The percentage and mass of each fatty acids (FA) was calculated using the peak area of the samples corrected with the respective correction factors.

### 2.3. Preparation and Analysis of Microcapsule

#### 2.3.1. Emulsion Preparation

The emulsion wall material formulations were thoroughly dissolved in deionized water at 60–70 °C and stored at 4 °C overnight for rehydration (Table 1). The oil extracted from *L. barbarum* was consequently administered gradually and prehomogenized through shear homogenizer employment (ULTRA-TURRAX IKA T18 Basic^®^, IKA, Schwarzwald, Germany) for 5 min at 5000–7000 rad/min. The total solid content was 30%. Emulsions were prepared using a final two-step homogenization at 20–30 MPa in a high-pressure homogenizer (SAMRO HOMOGENIZER^®^, Shanghai, China). The emulsion was consequently utilized as the feed liquid for the following spray-drying procedure.

#### 2.3.2. Spray-Drying Procedure

All emulsions were subjected to a B-290 mini spray-dryer for the spray-drying procedure (Büchi Labortechnik AG, Flawil, Switzerland) (0.7 mm nozzle and 60 mm × 50 mm × 110 mm main spray chamber). The flow rate and inlet air temperature could be optimized in order to maintain a constant outlet temperature of 90 °C ± 5 °C. All successfully spray-dried samples were collected and kept at 4 °C.

#### 2.3.3. Microencapsulation Efficiency

The amount of surface oil was measured according to previously described methods [18]. Briefly, 1 g of microencapsulated powder was added with 20 mL of light petroleum ether (60–80 °C) in an Erlenmeyer flask with a stopper and stirred at 25 °C in the dark for 15 min. The solvent mixture was passed through a Buchner funnel containing a filter paper, then collected and evaporated using a rotary evaporator in a water bath at a temperature of <30 °C to minimize the influence of heating on lipid oxidation. The amount of surface oil was calculated based on the difference between the initial clean flask and that containing the extracted oil residue. The amount of surface oil was calculated based on the difference between the initial clean flask and a flask containing the extracted oil residue.

Pont’s method was utilized to calculate overall oil yield from the spray-dried micro-capsules [19]. Briefly, 10 g of the powder was mixed with 20 mL of water at 50 °C in an Erlenmeyer flask with a stopper. After adding 15 mL of a de-emulsification reagent, the mixture was shaken vigorously and left to stand in a 70 °C water bath for 6 min. The resulting mixture was then centrifuged at 3000× *g* for 10 min, and the total oil was collected. For preparing the de-emulsification reagent, 10 g of sodium salicylate and 10 g of sodium citrate were dissolved separately in double-distilled water, followed by mixing these solutions together with 18 mL of n-butanol, and the volume was increased to 90 mL using double-distilled water. The following equation was used to calculate the microencapsulation efficiency (MEE):

MEE% = ((Total oil − Surface oil) ×100)/Total oil(3)

#### 2.3.4. Moisture Content

Powders’ moisture content was determined gravimetrically by drying in a vacuum oven at 70 °C until reaching a constant weight [20].

#### 2.3.5. Wettability and Solubility

The wettability of the powders was determined using the method described by Fuchs et al. [21]. One gram of powder was sprinkled over the surface of 100 mL of distilled water at 20 °C without agitation. The time taken for the powder particles to sediment, sink, be submersed, and disappear from the water’s surface was recorded and used for a comparison of the extent of wettability of the samples.

The solubility of the powders was evaluated according to the method proposed by Cano-Chauca et al. [22], with modifications. The powders were weighed (1 g) and stirred into 25 mL of distilled water for 5 min using a blender. The solution was then centrifuged at 3000× *g* for 10 min. An aliquot of 20 mL of the supernatant was transferred to a preweighed petri dish and oven-dried at 105 °C overnight. The solubility (%) was calculated as the percentage of dried supernatant in relation to the amount of powder originally added.

#### 2.3.6. Scanning Electron Microscopy (SEM) Analysis

Scanning electron microscopy (1.0 kV) was utilized in order to investigate any microcapsule microstructural issues (Leo Electron Microscopy Ltd., Cambridge, England, UK) [23]. Powder samples were attached to a two-sided adhesive tape mounted on the microscope stubs, and redundant powder samples were removed. The microscope was operated at an accelerated voltage of 1.0 kV.

#### 2.3.7. Accelerated Storage Test

All sample powders (in airtight glass containers) were stored at 4 °C and 60 °C for approximately 6 weeks in order to evaluate microencapsulated oil oxidative stability properties during this timeframe. This property was measured on a weekly basis through attaining peroxide values (POVs) of oil aliquots (1 g) from each powder. All unencapsulated oils were additionally stored and investigated in the same manner.

POVs were attained in line with a previously described method, albeit with minor modifications [24,25]. A 250 mL Erlenmeyer flask was employed for containing the collected oil, the latter being dissolved in a 50 mL volume of acetic acid/chloroform (3:2) formulation, followed by the addition of 1 mL of potassium iodide and consequent mixture-shaking for 60 s while the flask was sealed (rubber stopper). The resulting formulation was placed to rest for 3 min in a dark area, followed by dilution with 30 mL of water and consequent titration with 0.001 mol/L sodium thiosulphate (Na_2_S_2_O_3_) until near-total absence of yellow coloration was achieved. A 1 mL aliquot of 5 g/L starch solution was consequently added to the solution and re-titrated with Na_2_S_2_O_3_ (same concentration as before) until absence of blue coloration in the mixture was attained (end of reaction). All analyses were conducted in triplicate, together with a negative control/blank sample under equivalent conditions.

### 2.4. Statistical Analysis

All analyses were run in triplicate, and all results given in the tables and figures are expressed as mean ± standard deviation. Differences between the two systems were tested for significance by one-way analysis of variance using a statistical analysis system (IBM SPSS 22.0 for Windows, SPSS Inc., Chicago, IL, USA). The difference was considered significant at a level of *p* < 0.05.

## 3. Results and Discussion

### 3.1. SFE-CO_2_ Extraction

#### 3.1.1. SFE-CO_2_ Extraction Condition Optimizations

In this experiment, the seeds and peels of *L. barbarum,* which were from juice processing, were crushed to an 80–100 mesh size and dried at 55 °C, and the moisture content of raw material was detected to reach 6.20 ± 0.33%.

Extraction was performed under varying ranges for pressure, temperature, flow rate of CO_2_, and extraction time, together with the degree of ethanol addition as a modifier. In all extraction analyses, the recovered extract was a red-colored oil.

**Figure 1:**
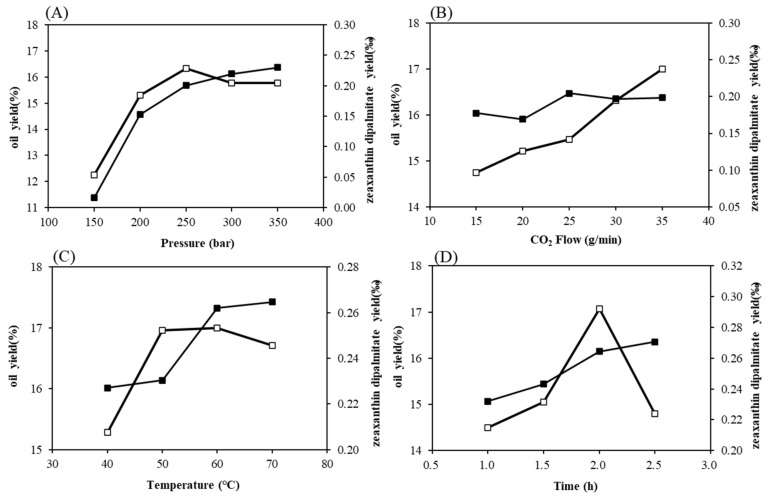
Effect of extraction pressure (**A**), CO_2_ flow (**B**), temperature (**C**), and dynamic extraction time (**D**) on the oil yield (□) and zeaxanthin dipalmitate yield (■).

Extraction pressure is an important factor affecting the yield of oil. As shown in Figure 1A, oil yields were increased with increasing pressure from 150 to 350 bar. There was a positive correlation between pressure and oil yield. The yield of zeaxanthin dipalmitate was increased with increasing pressure until 250 bar, when it reached the maximum. Because increasing the extraction pressure at a fixed temperature of 60 °C, a CO_2_ flow of 40 g/min, and a timespan of 2.0 h led to a higher fluid density, the solvent strengthened, which increased the solubility of the analytes. However, when the pressure increased to a certain extent, the solubility of CO_2_ increased slowly, and the yield of pigment (zeaxanthin dipalmitate) increased little, or even decreased. Nonetheless, when taking into consideration the overall oil yield and the extraction content of zeaxanthin dipalmitate, as well as the health/safety variables, potential loss of equipment, and energy consumption, a pressure of 250 bar was selected as optimum [26,27].

Due to increased CO_2_ flow, at a fixed temperature of 60 °C, a pressure of 250 bar, and a timespan of 2.0 h, the mass-transfer impetus and coefficient could be correspondingly increased, so that the mass-transfer rate could be accelerated, and the extraction capacity of SFE-CO_2_ was improved accordingly. Therefore, when the CO_2_ flow rate was 35 g/min, the oil yield reached about 17%. However, excessive CO_2_ flow increased the flow rate of CO_2_ in the extractor, shortened the residence time of CO_2_, and reduced the contact time with the extracted material, which was not conducive to improving the pigment extraction efficiency rate. Therefore, with the increase of CO_2_ flow, the yield of zeaxanthin dipalmitate remained at a certain value. Considering the overall oil yield and the extraction content of zeaxanthin dipalmitate, a CO_2_ flow rate of 30 g/min was selected to be optimal in this study (Figure 1B).

For temperature, at a fixed CO_2_ flow of 30 g/min, a pressure of 250 bar, and a timespan of 2.0 h, the extraction yields of the oil and zeaxanthin dipalmitate initially increased to a peak-maximum at 60 °C, as demonstrated in Figure 1C. This observation was most possibly due to temperature fluctuations having an influence on the analyte solubility/fluid density, whereby any temperature increases above 60 °C did enhance analyte volatility properties, but were detrimental to supercritical CO_2_ density levels. By increasing the temperature, the volatilities of the analytes could be increased, but the supercritical CO_2_ density decreased. So, when the temperature rose to 65 °C, the extraction yield of oil dropped to about 16.5%. Considering the overall oil yield and the extraction content of zeaxanthin dipalmitate, the optimal extraction temperature was 60 °C.

In Figure 1D, at a fixed temperature of 60 °C, a CO_2_ flow of 30 g/min, and a pressure of 250 bar, we found that with the increase of extraction time, the oil yield increased markedly and reached the maximum of 17% at 2.0 h, and then decreased sharply. This was because with the extension of the extraction time, the CO_2_ fluid fully contacted the raw material, the affinity for the extract increased, and the oil yield increased. However, with the prolonging of extraction time, part of the seed oil dissolved in the fluid resulted in an increase of CO_2_ viscosity and poor fluidity, leading to the decrease of extraction efficiency. With the prolonging of extraction time, the yield of pigment increased gradually, and more, the increase of yield of pigment decreased and tended to be balanced at about 0.26‰. Considering the overall oil yield and the extraction content of zeaxanthin dipalmitate, the optimal extraction time was 2.0 h.

Overall, the extraction yield was enhanced through optimization of temperatures, pressures, and times of extraction. One observation was that the cosolvent had a significant influence on the extraction of *L. barbarum* seeds and peels. Through adding 2% ethanol as the cosolvent for SFE-CO_2_ extraction, the final oil yield was reduced by approximately 10%, and the yield of zeaxanthin dipalmitate was increased by approximately twofold, which implied that the quality of the extracted core target (zeaxanthin dipalmitate) had increased by approximately twofold (Table 2).

Finally, the optimum extraction conditions were obtained, and they were as follows: extraction pressure, 250 bar; extraction temperature, 60 °C; dynamic extraction time, 2.0 h; and CO_2_ flow, 30 g/min (Table 2). The yields of oil and zeaxanthin dipalmitate under these conditions were 17.0 and 0.08 g/100 g, respectively (expressed as g/100 g seeds and peels).

#### 3.1.2. Analysis of Fatty-Acid Composition and Zeaxanthin Dipalmitate

The seed oil of *L. barbarum* that was extracted using SFE-CO_2_ at optimal conditions contained a high level of fatty acids (FAs) required by the human body. The saturated fatty acids were primarily palmitic acid (C16:0) and stearic acid (C18:0), this being approximately 8.15 ± 0.27% of the total content. The unsaturated fatty acids (UFAs) were primarily linoleic acid (C18:2), oleic acid (C18:1), and γ-linolenic acid (C18:3), with their contents being as high as 91.85 ± 0.27%, where linoleic acid (C18:2) demonstrated the highest concentration (430.26 ± 8.12 mg/mg, 65.18 ± 0.89%), followed by oleic acid (C18:1; 146.02 ± 5.20 mg/mg, 22.12 ± 0.75%; Table 3). It is known that UFAs can effectively control the levels of serum cholesterol and triglycerides, promote the development of the cerebral nervous system, and exert the functions of preventing cell senescence and cancer. Linoleic acid, an essential FA, plays a vital role in regulating human immunity, eliminating inflammation and inhibiting the synthesis of blood lipids. Therefore, based on the results of these FAs, *L. barbarum* can possibly be considered as a beneficial and novel dietary source for such essential FAs. Emerging scientific evidence also suggests that increased consumption of omega-3 FAs is associated with reduction of cardiovascular disease risks [28].

**Figure 2:**
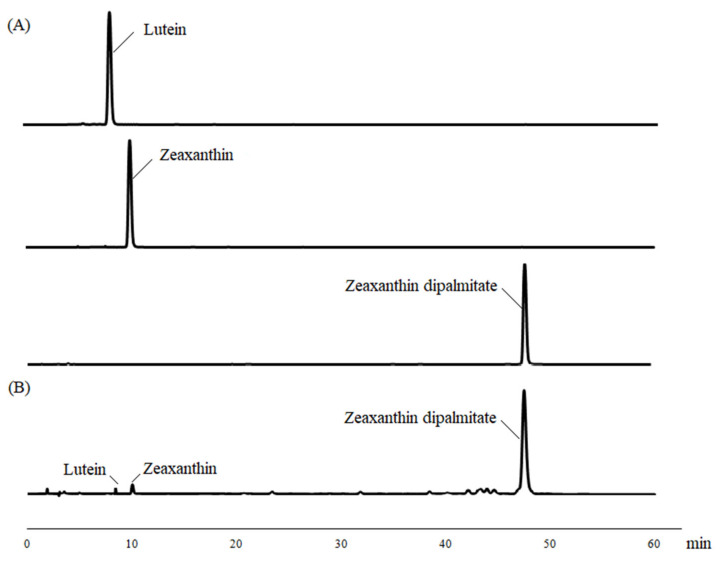
(**A**) High Performance Liquid Chromatography (HPLC) chromatogram of standards of lutein, zeaxanthin, and zeaxanthin dipalmitate, and (**B**) the extraction of *L. barbarum* seeds and peels by SFE-CO_2_.

Figure 2 shows HPLC chromatograms of standards of lutein, zeaxanthin, and zeaxanthin dipalmitate and the extraction of *L. barbarum* seeds and peels by SFE-CO_2_. The retention times of lutein, zeaxanthin, and zeaxanthin dipalmitate in the extracts were 8.05, 9.85, and 47.86 min, respectively. Standard solutions containing 10–100 μg were used to establish the standard calibration curves, which were linear and reproducible. All the correlation coefficients (R^2^) were above 0.999. Regarding the proposed *L. barbarum* seed oil, as depicted in Figure 2, the content of zeaxanthin dipalmitate was the highest of all carotenoids, with the concentration reaching 1877.48 ± 23.15 mg/mL. This comprised >85% of the total carotenoid content, and the extraction yield reached 0.85‰. The seed oil also contained small amounts of zeaxanthin, lutein, and other carotenoids, which accounted for 15% of the total carotenoid content. It has been reported that zeaxanthin dipalmitate was a highly prevalent carotenoid, forming 31–56% of the overall carotenoid content found in *Fructus lycii* [6].

### 3.2. Microcapsule Preparation

#### 3.2.1. Powder Characteristics

Moisture content is an important factor during microcapsule formation and affects drying properties, powder-flow capacity and long-term storage stability due to moisture-directed influences on crystallization properties and glass transition. Overabundant moisture levels lead to powder agglomerations and mildew development, consequently paving the way for oil release and oxidative destabilization. In conformity with past studies on this matter, the moisture content levels across the five microcapsule study groups ranged between 1.98 ± 0.05% and 2.79 ± 0.09% (Table 4). The moisture content of the microcapsule made of octenylsuccinate starch (OSA-starch), gum arabic (GA), and maltodextrin (MD) (No. 4) was the lowest. However, albeit negligible, such a moisture content variation could have been induced by the unique chemical composition of the varying barrier materials.

The moisture-content readings identified in this study were remarkably similar to findings from other investigations in this research niche. Microencapsulation measures for oregano essential oil through spray-drying with OSA-starch, GA, and MD as barrier materials is such an example, with moisture content in the range of 1.30–3.65% [29].

The wettability, or ability to absorb water, of microcapsules is one of the most important physical properties related to reconstituting the powders, and it is directly affected by the molecular interactions between the two phases [30]. The wetting times taken for the powders of OSA-starch and OSA-starch:GA:MD were 256 ± 5 and 298 ± 11 s, respectively. This indicates that OSA-starch, having a more hydrophilic character than does GA and MD, increased hydrophilicity of the wall systems, thus facilitating the accessibility and penetration of water into the powder particles. However, the wetting time taken for the powders of OSA-starch: Soy isolate protein (SPI) was 485 ± 12 s. This showed that SPI had a certain hydrophobicity.

To be practical, powders used as ingredients for the food industry must exhibit good solubility. Solubility is the last particle-dissolution step and is a decisive factor for the quality of these products [31]. All of the powders were relatively soluble despite the hydrophobic nature of the core material, yielding results ranging from 52.13 ± 0.16% to 66.22 ± 0.24% (Table 4). The solubility of powders made of OSA-starch:GA:MD (No. 4) was the highest. Pure *L. barbarum* seed oil was not soluble in pure water at room temperature, whereas encapsulating the essential oil resulted in better solubility. The type of encapsulant used did not affect this property.

Microencapsulation efficiency is considered to be paramount for attaining oxidation stability and consequent long-term storage integrity maintenance for essential oils. As demonstrated in Table 4, the encapsulation efficiency in this study varied between 68.85 ± 0.25% and 92.83 ± 0.13%. The MEE of powders made of OSA-starch:GA:MD (No. 4) was the highest. Starches are widely utilized in the food industry, since they are adept in film-layer development, consequently facilitating microencapsulation with reduced oil-handling losses. In addition, gum arabic has excellent volatile-compound retention and emulsification properties. Modified/adapted starches also possess such qualities, albeit to a lower extent than gum arabic [32]. A great example of a high-specification barrier material is a hydrolyzed starch, such as maltodextrin, which is widely utilized for microencapsulation of foodstuffs in the food industry [33]. Maltodextrin’s advantageous properties include its inexpensive production costs, bland taste/aroma, minute viscosity when concentrated in solid format, and oxidation resistance. However, maltodextrin suffers from a low emulsification propensity. Consequently, maltodextrin should always be utilized as part of a combinatory mixture containing surface-active biopolymers as well, such as gum arabic [34,35] or modified starches [36], to achieve effective microencapsulation with spray-drying.

#### 3.2.2. Morphology

**Figure 3:**
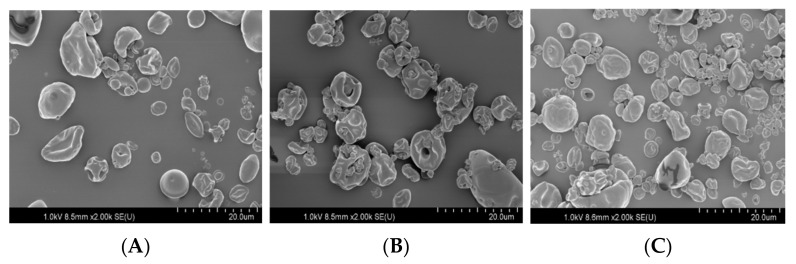
Scanning electron micrographs of the particles containing *L. barbarum* oil using the following wall materials: (**A**) OSA-starch, (**B**) OSA-starch/GA/MD, (**C**) OSA-starch/GA/LPS. OSA-starch: octenylsuccinate; GA: gum arabic; MD: maltodextrin; LPS: *L. barbarum* polysaccharide.

The SEM images (Figure 3) did not highlight any signs of particle cracking in all three microencapsulating mixtures. This is essential in order to maintain low gas permeability and consequently provide increased protective cover for *L. barbarum* residue oil. Each particle type exhibited consistent surface features of a spherical format. Spray-drying induces such particles to exhibit concave/shriveled surface features. In all cases, the powders existed as highly agglomerated minute particles. The microcapsules prepared using only OSA-starch exhibited a rounded external surface containing characteristic concavities (Figure 3A). The microcapsules prepared using OSA-starch solely, or utilizing starch and maltodextrin, had a higher proportion of spherical-shaped particle infrastructure, possibly due to enhanced elasticity properties exhibited by such combination mixtures when exposed to drying procedures.

#### 3.2.3. Storage Stability of Microcapsules

POV, a measure of the amount of hydroperoxide in a sample, represents the initial stage of oil deterioration, and is a standard index used to monitor food safety and quality [37]. In general, products have a lower solid content and higher oil concentrations, which can result in the presence of oil on the particle surface. This can be related to the lower encapsulation efficiency obtained under these conditions, which produces higher peroxide values. When this unencapsulated oil contacts oxygen, it is much more susceptible to oil oxidation than its encapsulated counterpart [38].

**Figure 4:**
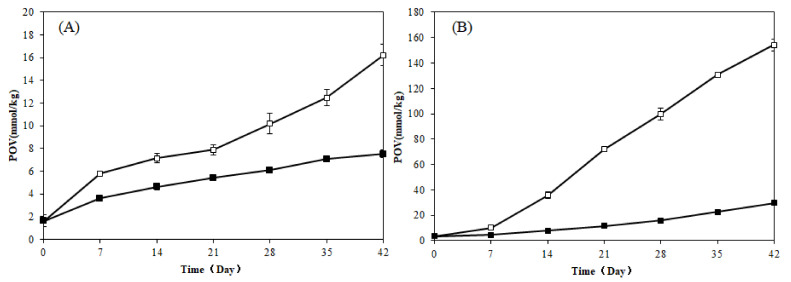
The peroxide value (POV) changes at 4 °C (**A**) and 60 °C (**B**) for 6 weeks of the free oil (□) and microencapsulated powder (■).

POV changes were measured at different temperatures (4 °C and 60 °C) to evaluate the stability of microcapsule oil. At day 0 before storage, all oil samples had an initial POV of approximately 2.00 mmol/kg oil. No significant differences were observed between the encapsulated and control oils at day 0, which implied that lipid oxidation did not occur during the encapsulation process (i.e., emulsion preparation and spray-drying). As illustrated in Figure 4A, the POV of zeaxanthin dipalmitate-rich oil was 16.23 ± 0.13 mmol/kg after 6 weeks, and the microcapsules exhibited no significant increase in peroxide values during storage at 4 °C. Conversely, when the storage temperature was 60 °C for 6 weeks, the zeaxanthin dipalmitate-rich oil exhibited a maximum peroxide value of 154.09 ± 4.63 mmol/kg (Figure 4B), whereas that of the microcapsule product was approximately 29.81 ± 0.58 mmol/kg, confirming that the microcapsule products exhibited efficient heat-resistance performance and helped protect the oil quality. In contrast, the POV of unencapsulated *L. barbarum* seed oil rapidly accelerated with increasing temperatures. The seed oil under the protection of the capsule wall could not only effectively be isolated from entry of oxygen, it also was protected from volatilization, which could significantly extend the shelf life of the oil.

## 4. Conclusions

This study demonstrated how extraction temperatures, pressures, timing, CO_2_ flow rates, and cosolvents can influence the extraction of *L. barbarum* seed oil and the final yields of oil and zeaxanthin dipalmitate for complete extraction. Addition of ethanol significantly increased the final yield of zeaxanthin dipalmitate. The recovered extract was a red-colored oil that was subsequently microencapsulated by spray-drying using OSA-starch, gum arabic, and maltodextrin as a combined wall formulation to avoid the degradation of lipids over the storage time. Results indicated that these wall materials exhibited the highest microencapsulation efficiency ever registered in such studies (92.83 ± 0.13%), with a moisture content and solubility of 1.98 ± 0.05% and 66.22 ± 0.24%, respectively. The peroxide value of microencapsulated zeaxanthin dipalmitate-rich oil remained at one-ninth of that of unencapsulated oil after accelerated oxidation at 60 °C for 6 weeks, thus revealing the promising oxidation stability of zeaxanthin dipalmitate-rich oil in microcapsules.

## Figures and Tables

**Table 1:** Composition of the wall materials for each treatment used as a feed solution for the spray-drying process.

No.	Wall Material (g 100 g^−1^ of Solution)					Core Material (g 100 g^−1^ of Solution)	Solid Content (%)
	OSA Starch (OSA)	Gum Arabic (GA)	Maltodextrin (MD)	Soy Isolate Protein (SPI)	*L. barbarum* Polysaccharide (LPS)	*L. barbarum* Seed Oil	
1	24.0					6.0	30.0
2	12.0	12.0				6.0	30.0
3	12.0			12.0		6.0	30.0
4	13.5	7.5	3.0			6.0	30.0
5	13.5	7.5			3.0	6.0	30.0

**Table 2:** The optimal supercritical fluid CO_2_ extraction (SFE-CO_2_) conditions and yield of zeaxanthin dipalmitate and oil.

Extraction Conditions	Optimum	Zeaxanthin Dipalmitate Yield (‰)	Oil Yield (%)
Extraction pressure	250 bar	0.26 ± 0.05 ^a^	17.0 ± 0.67 ^a^
Extraction temperature	60 °C		
Dynamic extraction time	2.0 h		
CO_2_ flow	30 g/min		
Cosolvent	2% ethanol	0.85 ± 0.11 ^b^	15.2 ± 0.42 ^b^

^a^ Optimal conditions without cosolvent. ^b^ Optimal conditions with cosolvent.

**Table 3:** The content of fatty acids (FAs) in the oils from *L. barbarum* seeds and peels.

FAs	mg/mg (oil) ^a^	Relative Content (%) ^b^
Linoleic acid (C18:2)	430.26 ± 8.12	65.18 ± 0.89
Oleic acid (C18:1)	146.02 ± 5.20	22.12 ± 0.75
γ-Linolenic acid (C18:3)	30.04 ± 1.81	4.55 ± 0.30
UFAs	606.32 ± 6.61	91.85 ± 0.27
Palmitic acid (C16:0)	21.52 ± 1.15	3.26 ± 0.20
Stearic acid (C18:0)	32.28 ± 0.33	4.89 ± 0.07
SFAs	53.80 ± 1.41	8.15 ± 0.27
TFAs	660.12 ± 5.47	100 ± 0.00

FAs: fatty acids; UFA: unsaturated fatty acids; SFA: saturated fatty acids; TFA: total fatty acids. ^a^ Absolute content (mg/mL, mg (FA)/mL(oil)). ^b^ Relative content (%, ratio of the content of one FA with that of all FAs).

**Table 4:** Mean values and standard deviations for the moisture content, wettability, solubility, and microencapsulation efficiency (MEE%) of powders produced.

No.	Wall Material	Variables			
		Moisture (%)	Wettability (s)	Solubility (%)	MEE (%)
1	OSA-starch	2.02 ± 0.17 ^a^	256 ± 5 ^a^	64.96 ± 0.35 ^a^	68.85 ± 0.25 ^a^
2	OSA-starch:GA	2.33 ± 0.22 ^b^	301 ± 8 ^b^	62.65 ± 0.37 ^b^	82.22 ± 0.18 ^b^
3	OSA-starch:SPI	2.79 ± 0.09 ^c^	485 ± 12 ^c^	55.12 ± 0.45 ^c^	76.92 ± 0.05 ^c^
4	OSA-starch:GA:MD	1.98 ± 0.05 ^a^	298 ± 11 ^b^	66.22 ± 0.24 ^d^	92.83 ± 0.13 ^d^
5	OSA-starch:GA:LPS	2.59 ± 0.16 ^c^	322 ± 10 ^e^	52.13 ± 0.16 ^e^	83.92 ± 0.24 ^b^

OSA-starch: octenylsuccinate; GA: gum arabic; MD: maltodextrin; SPI: soy isolate protein; LPS: *L. barbarum* polysaccharide. Different small letter superscripts (^a–e^) denote the significant difference (*p* < 0.05).
